# Long-term survival in lung transplant recipients depending on icu length of stay: outcome in a series of 653 consecutive patients

**DOI:** 10.1186/2197-425X-3-S1-A450

**Published:** 2015-10-01

**Authors:** M Zemtsovski, M Østergaard, P Bredahl, M Perch, C Møller, M Iversen

**Affiliations:** Department of Cardiothoracic Intensive Care, Copenhagen University Hospital - Rigshospitalet, Copenhagen, Denmark; Department of Cardiology, Division of Lung Transplantation, Copenhagen University Hospital - Rigshospitalet, Copenhagen, Denmark; Department of Cardiothoracic Surgery, Copenhagen University Hospital - Rigshospitalet, Copenhagen, Denmark

## Introduction

Over the last two decades lung transplantation (LT) has become the treatment of choice for various end-stage lung diseases. It is well-known that the majority of patients who underwent LT experienced complications postoperatively. Postoperative mortality has decreased due to improved surgical techniques and intensive care. However, very little is known about the long-term outcome of lung transplant recipients with prolonged intensive care length of stay (ICU LOS).

## Objectives

To determine long-term survival after LT depending on ICU LOS.

## Methods

A retrospective review of all patients receiving LT in Denmark in 1992-2014. The population of lung transplant recipients was divided into groups with respect to ICU LOS: 7, 14 and 28 days. Kaplan-Meier analysis was used to compare the overall survival between groups.

## Results

Lung transplantation was performed in 653 patients. Single lung transplantation was performed in 330 cases, double lung transplantation in 323 cases. Main indications were chronic obstructive pulmonary disease (n = 272), alpha1-antitrypsin deficiency (n = 141), cystic fibrosis (n = 99), pulmonary fibrosis (n = 67), sarcoidosis (n = 29), primary pulmonary hypertension (n = 14), and other end-stage lung diseases (n = 31).

The median ICU LOS was 3 days with interquartile range 5 and minimum-maximum 0-156 days. The median survival time (MST) for the entire series was 6.1 years.

**Figure 1:**
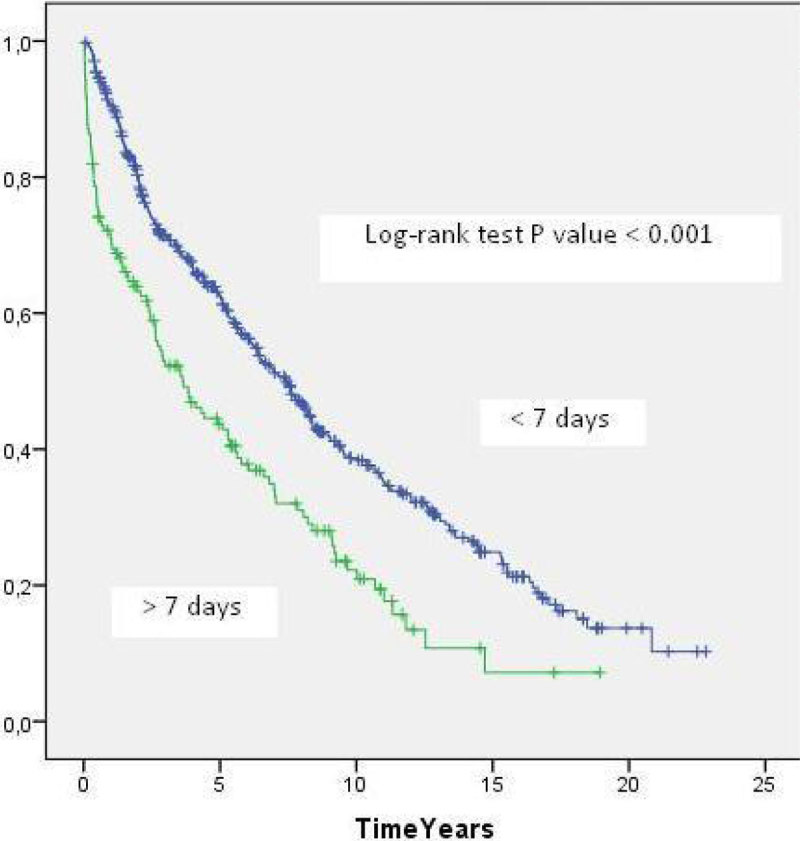
Figure 1

Of the 637 patients who survived the first 7 days after LT, 155 (24%) had ICU LOS more than 7 days. The MST in this group was 3.6 years compared with 7.4 years in the group of patients with ICU LOS ≤ 7 days (p < 0.001) (Figure 1).

**Figure 2:**
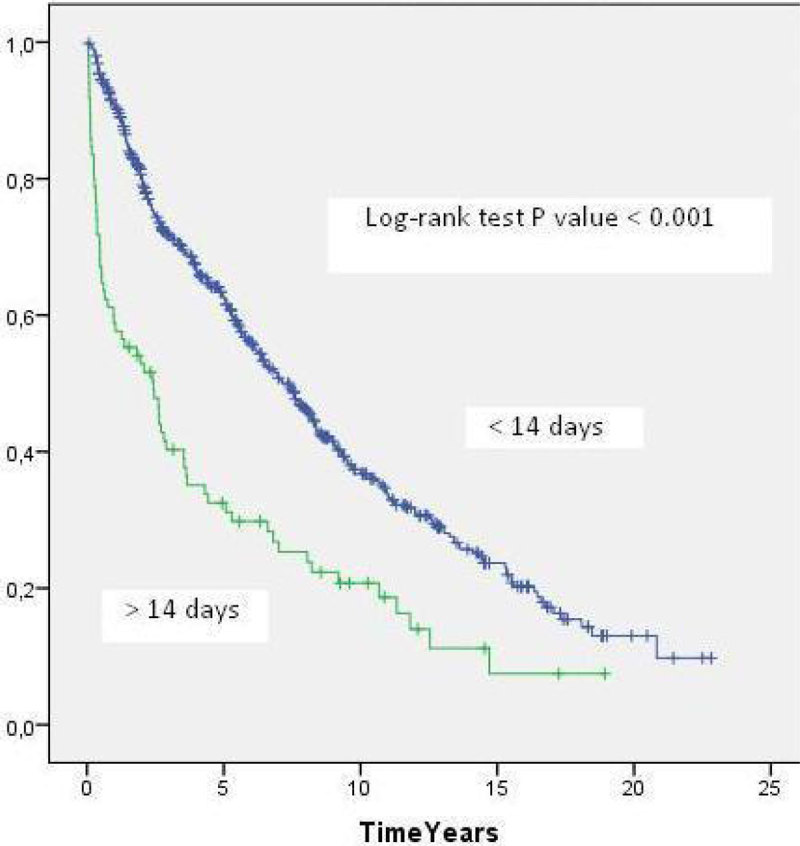
Figure 2

Of 631 patients who survived the first 14 days after LT, 85 (13%) had ICU LOS more than 14 days. The MST in this group was 2.4 years compared with 7.4 years in the group of patients with ICU LOS ≤ 14 days (p < 0.001)(Figure 2).

**Figure 3:**
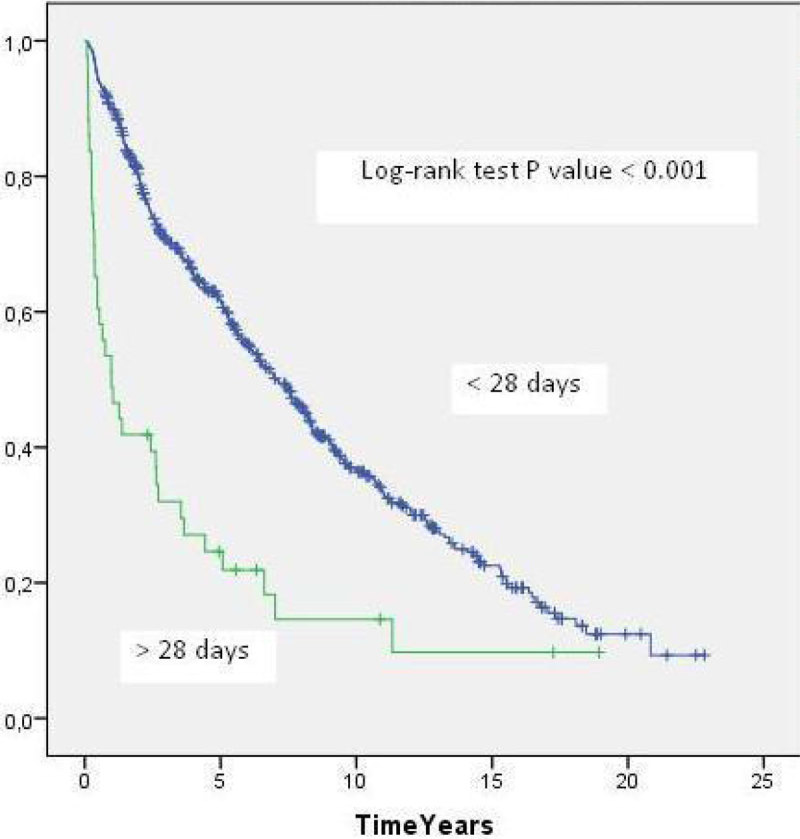
Figure 3

Of 614 patients who survived the first 28 days after LT, 43 (7%) had ICU LOS more than 28 days. The MST in this group was 1.0 year compared with 7.0 years in the group of patients with ICU LOS ≤ 28 days

(p < 0.001)(Figure 3.

## Conclusions

A significant number of LT recipients required prolonged ICU LOS postoperatively. The prolonged ICU LOS was associated with poor long-term survival.

